# The Brain–Heart Network of Syncope

**DOI:** 10.3390/ijms25136959

**Published:** 2024-06-26

**Authors:** Sailen Barik, Thomas Riddell

**Affiliations:** 1 Independent Researcher, EonBio, 3780 Pelham Drive, Mobile, AL 36619, USA; 2 Cardiology Associates of Mobile, Mobile, AL 36604, USA

**Keywords:** fainting, syncope, vagus nerve, neuropeptide Y, GPCR, adenosine receptors

## Abstract

Observed and recorded in various forms since ancient times, ‘syncope’ is often popularly called ‘fainting’, such that the two terms are used synonymously. Syncope/fainting can be caused by a variety of conditions, including but not limited to head injuries, vertigo, and oxygen deficiency. Here, we draw on a large body of literature on syncope, including the role of a recently discovered set of specialized mammalian neurons. Although the etiology of syncope still remains a mystery, we have attempted to provide a comprehensive account of what is known and what still needs to be performed. Much of our understanding of syncope is owing to studies in the laboratory mouse, whereas evidence from human patients remains scarce. Interestingly, the cardioinhibitory Bezold–Jarisch reflex, recognized in the early 1900s, has an intriguing similarity to—and forms the basis of—syncope. In this review, we have integrated this minimal model into the modern view of the brain–neuron–heart signaling loop of syncope, to which several signaling events contribute. Molecular signaling is our major focus here, presented in terms of a normal heart, and thus, syncope due to abnormal or weak heart activity is not discussed in detail. In addition, we have offered possible directions for clinical intervention based on this model. Overall, this article is expected to generate interest in chronic vertigo and syncope/fainting, an enigmatic condition that affects most humans at some point in life; it is also hoped that this may lead to a mechanism-based clinical intervention in the future.

## 1. Introduction

‘Syncope’ is popularly recognized as ‘fainting’ and is caused by a variety of conditions that can be metabolic (e.g., respiratory insufficiency, ionic, and hormonal imbalance), neurological, or situational (e.g., sad news, nervousness, vertigo, fear of heights, sight of blood, swallowing) [1,2,3,4,5,6,7,8]. In general, syncope (or vasovagal syncope) is a process by which the brain shuts down the less essential functionalities, such as vision, hearing, speech, volunteer motor movements, and neuron activities, and attains a deep sleep-like state, whereby only the basic metabolic rate (BMR) of the body is maintained, ensuring survival. It has been estimated that syncope hits one in three people at least once in their life, usually without any warning; although the elderly are more susceptible to syncope, it can occur in all age groups [1,2,3,4,5,6]. Barring very serious contributing factors, such as severe head injury or acute cardiac arrests, syncope is a reversible event, usually lasting only for a few seconds or minutes.

Syncope is manifested as the loss of a subset of functions of the brain, an energy-hungry organ that relies on an abundant supply of oxygen, provided by the cardiovascular system. Thus, the general consensus is that syncope can result from a functional deficiency of either the heart, the brain, or both. Even though syncope or transient fainting has attracted the attention of scientists and clinicians for ages [2,3,4,5,6,7,8], its exact mechanism remains obscure, mainly because most researchers specialize in either the heart or the brain in isolation but not in the interactions between them, which is clearly required to fully understand syncope [4,5,6,9,10,11].

## 2. The Early Recognition of Syncope and Hints to Its Mechanism

**Figure 1:**
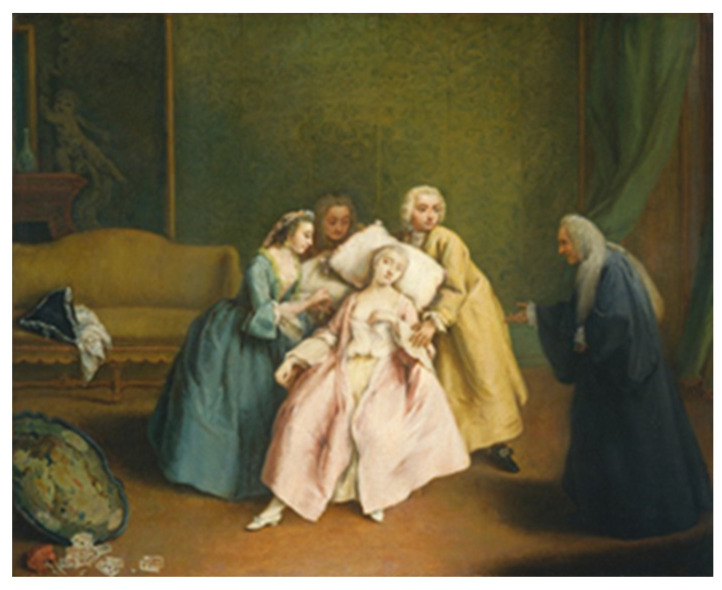
‘The Faint’ by Pietro Longhi, c. 1744. Source: National Gallery of Art, https://www.nga.gov/collection/art-object-page.204.html; accessed on 25 January 2024. (This is a faithful photographic reproduction of a two-dimensional, public-domain work of art, free of all copyrights).

Temporary loss of consciousness with recovery after varying periods has been recognized in human society for ages, as evidenced in stories and paintings, such as the famous painting, eponymously titled ‘The Faint’, by the Venetian artist Pietro Longhi (Figure 1) in 1744. The scientific foundation of syncope is closely related to the Bezold–Jarisch reflex [9], which is a triad of responses, namely apnea, bradycardia, and hypotension. Historic accounts of its discovery have been detailed in two early reviews [9,12] but will be briefly presented here in the context of syncope. The discovery goes back to Ludwig Friedrich Albert von Bezold, a German physiologist–scientist; while determining which nerve fibers are located in the spinal cord, he concluded that the heart is under constant vagus influence [12].

In a series of experiments, Bezold used the toxin veratrine, extracted from the roots of Veratrum album, and found that when injected into the jugular vein of a rabbit, it significantly reduced the blood pressure; interestingly, when both vagus nerves were severed, blood pressure and heart rate were promptly restored. Bezold correctly interpreted that afferent impulses originating in the heart traveled to the vasomotor center via the vagus. About 70 years later, Adolph Jarisch (1891–1965), an Austrian pharmacologist, rediscovered, confirmed, and extended the ‘Bezold effect’ to cats. He showed that veratrine induced a dramatic blood pressure depression in these animals. Like Bezold, he could reverse it by blocking the vagus nerve signal. He performed this, not by cutting the vagus nerve, as Bezold did, but by cooling the nerves, which allowed him to inquire about the reversibility of the effect. Indeed, after warming the vagus nerves, blood pressure decreased again, and this cycle could be repeated. The phenomenon was, therefore, both reversible and reproducible. In recognition of the two pioneers, this reflex came to be known as the Bezold–Jarisch reflex. Among other things, these studies, for the first time tied the cardio–pulmonary–vagal nerve axis to syncope.

### Types of Syncope

Syncope is a complex debility that may result from multiple excitatory and inhibitory homeostatic reflexes gone awry [3,8,13]. Currently, five major types of syncope have been recognized and are also distinguishable by their mechanism, although the distinction is not a trivial task, as there are substantial overlaps among them.

(i) Vasovagal syncope (also called neurocardiogenic syncope) is the most common form, constituting about half of all syncope cases. (ii) Situational syncope is actually a minor branch of vasovagal syncope. (iii) Postural or orthostatic syncope (synonymous with postural hypotension) is so called because it occurs from a quick change in position, such as standing up after lying down (sometimes described as ‘head up tilt’) [3]. It leads to a transient reduction in cerebral blood flow, and the loss of consciousness is very brief and low grade, sometimes lasting only for a second or two. (iv) Cardiac syncope is due to a defect in the heart or a blood vessel that reduces blood flow to the brain. (v) Neurologic syncope occurs when there is a neurological condition, such as a seizure or stroke. Other less common conditions include migraines and normal pressure hydrocephalus. Both neurologic and cardiac type syncopes are long-lasting problems, and hence require invasive or surgical help to reduce their occurrence, as described in Section 5.2 [14,15,16].

In this review, we have focused mainly on vasovagal syncope, not only for its prevalence but also because it can serve as a representative for essentially all other types due to the fundamental commonality between all syncopes, particularly in the final steps, namely, oxygen deficiency and the resultant shut down of the brain. Focusing on a single syncope has also allowed us to devote more attention and space to the common molecular signaling pathways.

## 3. The Basic Anatomy of Syncope

**Figure 2:**
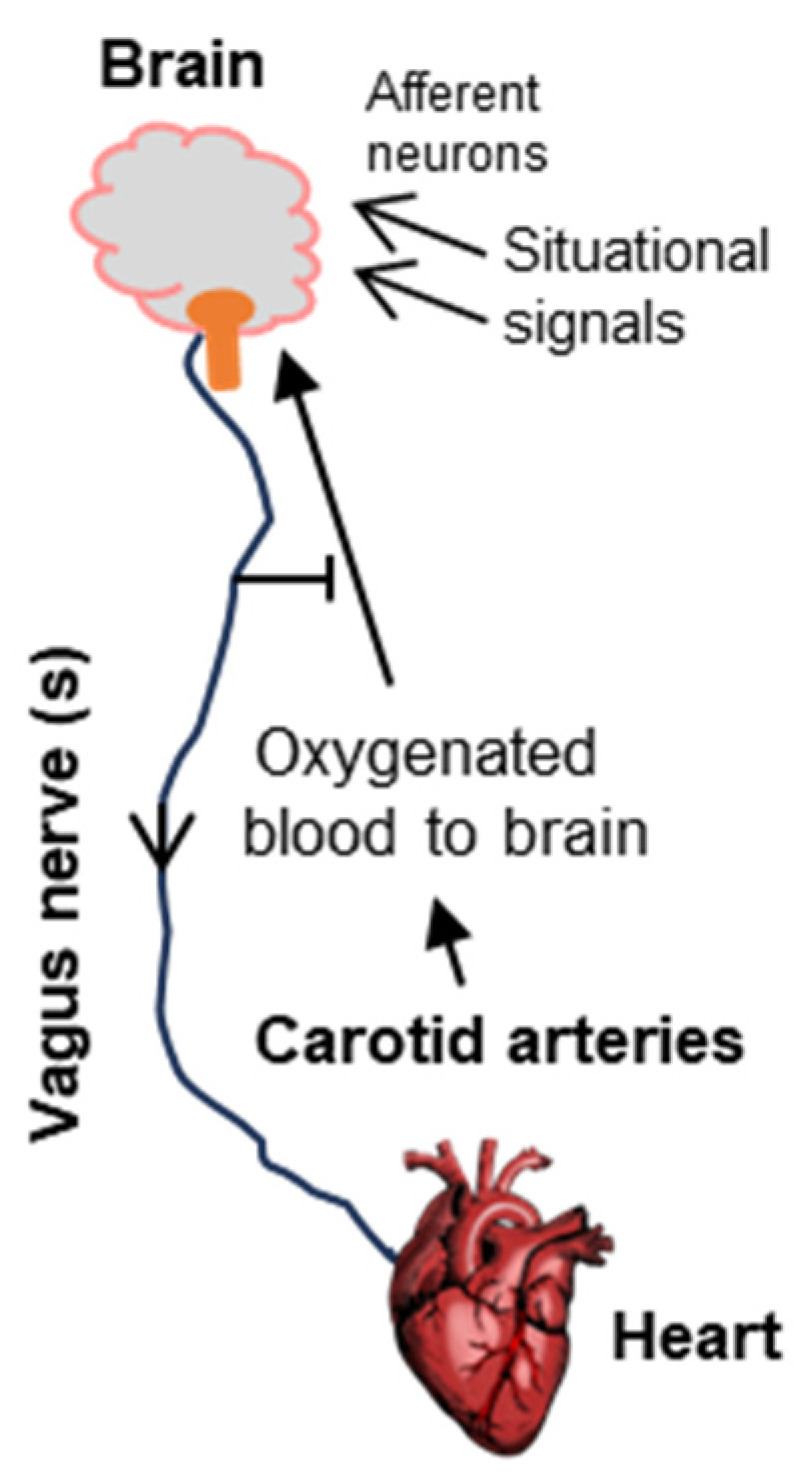
Schematic of the neurocardiologic axis, as inferred by Bezold and Jarisch, which has served as the foundational model of syncope. The organs and tissues are shown in bold fonts. Here, an overactive vagal nerve system, in its efferent role, reduces heart function to lower blood pressure, thus causing poor oxygen supply to the brain and fainting. The situational effectors (e.g., fear of heights, sight of blood, etc., Section 1) are diverse, generally carrying the cognate signal to the emotional area of the brain using afferent neurons, following which the efferent function of the vagus nerve again regulates the heart. Note that in its normal function, the vagus nerve plays an inhibitory role, preventing fainting (Section 2 and Section 3.1).

The Bezold–Jarisch research, described above, touched on the central signaling axis of syncope. The cardinal importance of the axis is underscored by the fact that syncope is often referred to as ‘vasovagal syncope’, whereas the sudden drop in heartbeat and blood pressure by direct stimulation of the vagus nerve is called “vagal response” (Figure 2). As we will see in the rest of the review, the latest model of syncope has been gradually built on this basic framework.

### 3.1. The Vagus Nerve

Since cognitive loss is the most visible outcome of fainting, the brain was always the first suspect. The Bezold–Jarisch research put the spotlight on the vagus nerve that originates from the brain and connects to the heart. The effect on the heart led to the regulation of oxygen supply to the brain, creating a circular and reversible regulatory loop. The loop also helps to explain how the fainting signals that directly affect the brain, such as visual cues, emotional stress, and head injury, can lead to fainting.

The term ‘vagus nerve’, although generally used in the singular, actually refers to a family of ten cranial nerves out of a total of twelve, which is why it is sometimes designated as cranial nerve X or CN X, for the Roman numeral ten. A full treatise of the vagus nerve family is out of the scope of this short review; however, interested readers may consult a multi-part comprehensive monograph by Yan and Silberstein, of which the first two parts have been cited here [17,18]. Collectively, the vagus nerves control a multitude of physiological functions, including heart rate, digestion, and breathing [10,19,20]. The particular vagus nerve involved in syncope is often referred to as vagal sensory neuron (VSN). In part due to the complexity of the vagus family, the molecular identity, anatomical and physiological characteristics, or functional details of the VSNs remained unknown for many years. This changed in 2023 with a series of ground-breaking experiments by Lovelace et al. [1]. These researchers used a combination of single-cell RNA sequencing (RNAseq) and a newly developed hydrogel-based reinforcement of a three-dimensional imaging technique (abbreviated HYBRiD) [21] to show that VSNs that express the neuropeptide Y receptor type 2 (NPY2R) are the ones that connect the heart ventricular wall to the area postrema. NPY2R is widely distributed in the mammal, serving a multitude of physiological roles [22]. Structurally, NPY2R is a G protein-coupled receptor (GPCR) with seven transmembrane helices and belongs to the Class A type of GPCRs [23,24,25]. The name NPY2R derives from the fact it is the natural receptor for neuropeptide Y (NPY), a 36 amino acid neuropeptide (and its fragment, amino acids 13–36) that serves as the agonist for this receptor and is involved in various processes in the nervous systems. All peptides in this family end with a tyrosine residue, hence the designation Y. When Lovelace and coworkers activated the NPY2R VSNs by optogenetic techniques in a strategically constructed mouse, the classic Bezold–Jarisch responses, namely, a fall in blood pressure, heart rate, respiration, and eye movement [26], were observed, and the mouse actually fainted. Simultaneous echocardiography and laser Doppler flowmetry also showed the responses characteristic of syncope. The role of the NPY2R VSNs was fully confirmed by their ablation, which specifically abolished these responses. Together, these results recapitulated the features of human syncope in a laboratory animal model that is now amenable to further investigations.

Rodent models, in general, have been extremely useful for our understanding of the human brain [5,27,28]. Though the exact ortholog of rodent VSNs in human remains to be identified, a recent study of a set of 2835 homologous genes, using a supervised machine learning approach, revealed mouse–human neuroanatomical correspondences [29]. This study provides early confidence that the mouse model of the VSN/NPY2R may also be operative in human syncope (and perhaps in other mammals), but this remains to be experimentally proven.

As detailed in the sections that follow (e.g., Section 3.2 and Section 4), the adenosine receptors also play a cardinal role in neuronal response in general and likely in vasovagal syncope as well. Like NPY2R, these receptors also contain GPCRs with seven transmembrane helices. In what follows, these GPCRs are described in relevant detail.

### 3.2. The GPCR Feature of A1/A2A Receptors and NPY2R

**Figure 3:**
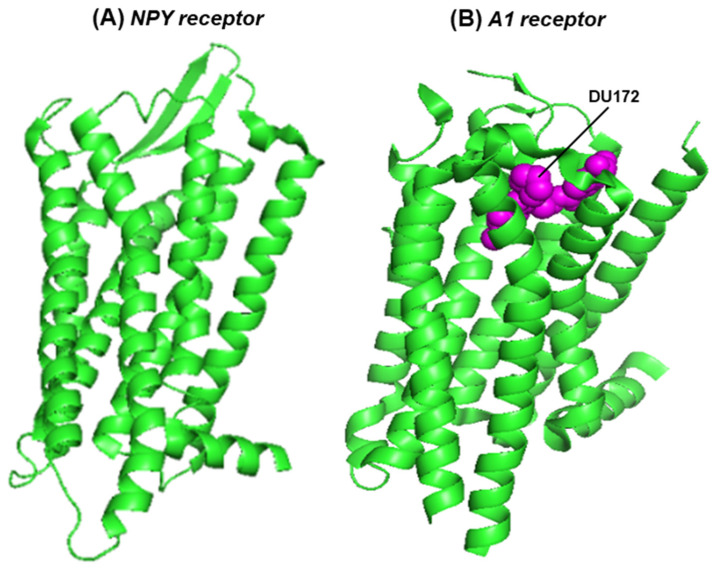
The seven-helix domain of two receptors is shown in a PyMOL presentation of the structures: (**A**) the NPY2R (crystal structure, PDB 7YOO) [24] and (**B**) the A1 receptor, in complex with the covalently attached, irreversible antagonist, DU172 (cryo-electron microscopy, PDB 5UEN) [31]. The protein structure is in the green ribbon presentation; DU172, cradled in the ligand-binding pocket, is represented in magenta-colored spheres.

G protein-coupled receptors (GPCRs) represent the largest protein family in the human proteome [28,29,30]. The seven transmembrane helices allow them to be embedded in the cell membrane and also form the ligand-binding pocket, whereas the allosteric modifiers of GPCRs bind at a separate pocket farther away. The adenosine receptors belong to the GPCR family, and adenosine is their natural ligand. The GPCRs, in general, exhibit high sequence diversity but share the conserved architecture (three-dimensional structure) of the seven-helix domain, within which the ligand-binding pocket is formed, whereby strategically placed conserved amino acid side chains bond with the ligand [25,31]. This pocket also binds agonists and structurally similar antagonists. The overall architectural similarity between the seven helical domains of the NPY2R and A1 receptors is shown here (Figure 3); the helical domain of the A2 receptor is very similar to that of A1, and, therefore, not shown here.

### 3.3. The Brain and the Heart

As mentioned earlier, the obvious loss of cognitive and motor functions in syncope implies that the brain–heart signaling network would eventually reach the brain to affect these functions. Unfortunately, the human brain, with its ~90 billion nerve cells, remains one of the least understood frontiers of human knowledge, and thus, much of its participation in syncope also remains unclear. Specifically, fine mapping of the structure–function network of the neurons has been a formidable task. Similarly, the molecular interaction between the VSN/NPY2R and the heart ventricular wall has not been defined. Nonetheless, we will present the areas where substantial progress has been made, which includes the mechanisms of signaling by NPY2R.

## 4. Molecular Signaling in Syncope

### 4.1. Adenosinergic Signaling

**Figure 4:**
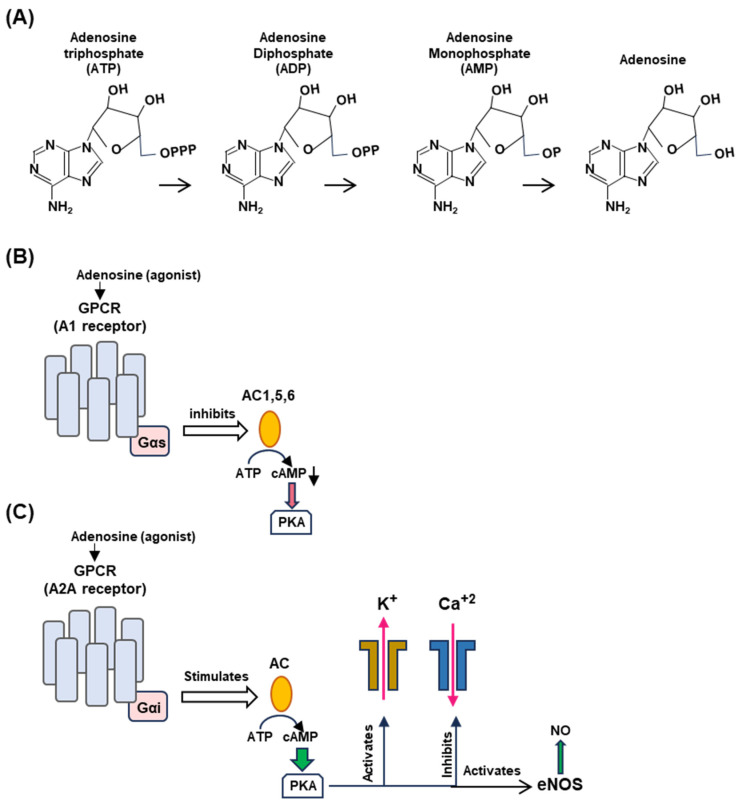
Generation of adenosine from ATP (**Panel A**) and differential regulation of A1 (**Panel B**) and A2A (**Panel C**) receptors. (**A**) Adenosine is produced by stepwise enzymatic dephosphorylation of ATP, the energy currency in living cells. The structures are presented in PubChem convention (https://pubchem.ncbi.nlm.nih.gov/, accessed on 10 February 2024). (**B**,**C**) Are highly schematic diagrams showing only the key players in the signaling cascade. In both panels, the seven-helix bundle represents the GPCR; the signaling starts with the engagement of the agonist, adenosine, or a pharmaceutical adenosine analog. The orange ellipse is adenylyl cyclase (also called ‘adenyl cyclase’ or ‘adenylate cyclase’; AC). For simplicity, we have indicated the stimulatory and inhibitory Gα subunits as Gαs and Gαi, respectively (pink rectangle), although they are in complex with other subunits. The guanine nucleotides, GDP/GTP, regulate the G proteins but are not shown here. (**Panel B**): When adenylyl cyclases 1, 5, and 6, are inhibited, cAMP synthesis is lowered, and so is PKA activation (indicated as red arrowhead). The effects on the potassium channel (stimulatory) and calcium channel (inhibitory) are shown in (**Panel C**) for the A2A receptor. (**Panel C**): The common symbols and shapes are described in (**Panel B**), but it is important to note the functional contrasts between the two. In the case of the A2A receptor, AC is stimulated (instead of inhibited) and PKA is fully activated (indicated by the wider green arrowhead), which then regulates the channels to release potassium and calcium ions and liberates vascular NO by activating eNOS. Signaling by the vagus nerve is not depicted here since the molecular aspects of signaling by the NPY2R GPCR, relevant to syncope, are not yet known.

Many of the molecular signaling mechanisms described here are based on known pathways of neuroendocrine signaling founded on the cumulative evidence from multiple complementary studies. Specifically, these studies indicated that syncope and related symptoms are in part due to an aberration of the adenosinergic system [32,33,34]. As mentioned before, the two receptors most implicated in NES (neuroendocrine syncope) are A1 and A2A [34], and hence, their locations in NES-relevant tissues will be focused on here (Figure 4) along with the salient features of the adenosinergic pathway. Note that this is a barebone schematic of the very elaborate adenosinergic system, and much of what is presented here is mostly based on the general pathway in normal cells and implicated for NES mainly by correlation or association. This is because of a number of reasons. (a) For ethical and technical reasons, human syncope cannot be replicated in human subjects. In fact, there is currently no bona fide primate model for a planned syncope study. (b) There is no tissue culture or in vitro cell-free system for a molecular dissection of the syncope response. Nonetheless, the overall associative evidence is substantial [33,34,35,36,37], and hence, it is presented here and in Section 4.2.

The differential effect of the adenosine receptors is dictated by multiple kinetic and regulatory parameters, such as their affinity for adenosine, as well as by tissue-specific locations [34,38,39]. The A1 receptor is coupled to the inhibitory Gα subunit that inhibits cyclases AC1, AC5, and AC6, thereby decreasing 3′,5′-cAMP production, whereas the A2A receptor is coupled to stimulatory Gα subunits that activate membrane-bound AC isoforms, thereby elevating the cAMP concentrations [Mosqueda-Garcia, Mark], stimulating cAMP-dependent signaling by activating cAMP-dependent protein kinase A (or protein kinase A; PKA) [10] (Figure 4B).

Activated A1R also causes activation of potassium channels but inhibits calcium channels [40,41]. A1R is expressed in the brain, the sinus, and atrioventricular nodes (SN, AVN) in the heart, thereby regulating both the heart rate and atrioventricular conduction. The A2A receptor, in contrast, is expressed in smooth muscle cells and induces dilation of the blood vessels through the triad of activation of potassium channels, the inhibition of voltage-gated calcium channels, and the activation of endothelial nitric oxide synthase (eNOS) via cAMP-activated PKA to promote NO synthesis (Figure 4C). Endothelial NO promotes vasodilation by relaxing the muscle tone. It should be reiterated that all molecules described in Figure 4 (viz. adenosine, GPCRs, cAMP, PKA, and NO) are involved in several smaller branches of NES and in scores of other signaling reactions outside NES that are outside the scope of the review; interested readers will easily find a large volume of research papers and reviews available on any of them, a few representatives of which are cited here [42,43,44,45,46,47,48,49].

### 4.2. Molecular Signaling Pathways of Syncope at the Organ Level

The two major organs involved in syncope are the heart and the head (Section 3.3). Using them as the main nodes and the signaling pathways presented above (Figure 4), we can present a molecular explanation for the fundamental features of syncope [34].

**Figure 5:**
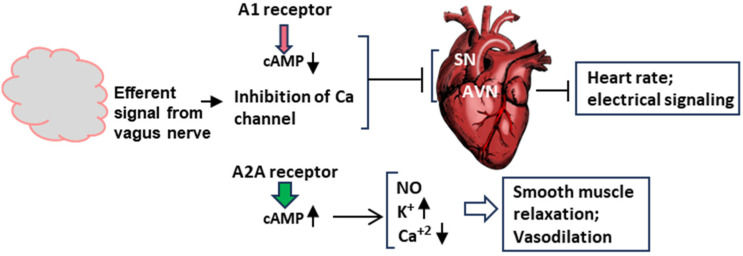
Schematic of the molecular signaling of syncope at the organ level. This diagram is a transposition of the signaling pathways in Figure 4 to the two major organs involved in syncope, namely, the brain and the heart. The vagus nerve is not shown here, but its signal contributes to the inhibition of the Ca channel. The action of the adenosine receptors, A1 and A2A, have been described earlier. In order to save space, this Figure and Figure 4 are complementary in terms of ion channels. The effect of A1 on the channels is shown here, and that of A2A is depicted in Figure 4 and Figure 5. SN and AVN are nodes inside the heart. Smooth muscle relaxation occurs both inside the heart and in blood vessels. Arrowheads pointing up mean increase, those pointing down mean decrease.

As illustrated here (Figure 5), in the early stages of syncope, fast A1 receptor signaling is mediated by the fast-moving neuroelectric signal of the vagal nerve from the brain, further activated by the traditional cholinergic mechanism using acetylcholine, leading to the inhibition of the Ca^+2^ channel. Subsequently, it is enhanced by the slower but more stable signaling by the cAMP pathway (Figure 4A). This promotes the hyperpolarization of the sinus (SN) and atrioventricular nodes (SN and AVN), causing a decrease in heart rate (clinically referred to as ‘negative chronotropy’) and electrical signal conduction in the heart (‘negative dromotropy’), hallmarks of vagal syncope.

In a concerted and complementary action, the A2A receptor signaling, working through the cAMP-dependent pathway, regulates the heart wall muscles (myocardium) so that the heart fails to generate enough output to send oxygenated blood to the brain (clinically, ‘inotropy’). Finally, along with the NO effect, the activation of voltage-regulated potassium channels and the inhibition of calcium channels leads to the relaxation of smooth muscle cells, facilitating vasodilation.

## 5. Potential Interventions Based on Known Signaling Pathways

### 5.1. Intervention via the GPCRs of Adenosine Receptors

In spite of the multiorgan complexity of syncope and the diverse role of the signaling molecules in many essential pathways of metabolism, some pharmacological therapy or prevention for syncope is available. The GPCR ligands, mainly the antagonists, comprise a major class of such compounds and will be elaborated here. We like to add that several members of this class have been also used for the treatment of other conditions, such as angina, pain, ischemia, and inflammation, and in imaging [49,50,51,52,53,54,55], underscoring the diverse roles of the large GPCR family. Here, we discuss a few representative compounds that have a mechanistic relationship with the signaling pathways of syncope described earlier.

**Figure 6:**
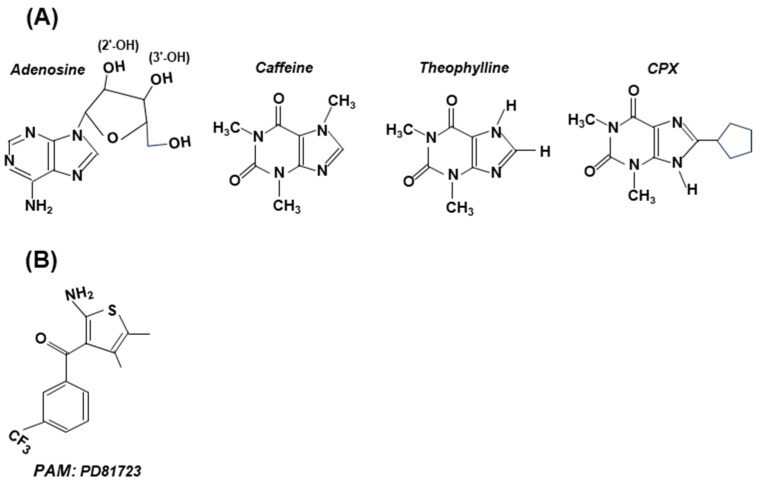
Structures of selected antagonists (with adenosine structure shown for comparison) (**Panel A**) and an allosteric modulator (PD81723) (**Panel B**). These are stick structures, drawn in two dimensions, and are not meant to be sterically accurate. (**A**) Note the presence of the adenosine’s ribose moiety in all antagonists, to which various substituents were added [51]. Depending on the amount, caffeine can be either stimulatory or inhibitory; both theophylline [57] and CPX have shown clinical promise. (**B**) PD81723 is a positive allosteric modulator (PAM), specific for A1. Note that its structure shows little resemblance to adenosine.

As a rule, the antagonists bear structural similarity to adenosine (Figure 6). Through the inhibition of the phosphodiesterase enzyme (PDE), caffeine (and other methylxanthines) also causes an intracellular increase in cyclic purine nucleotides, cAMP (as well as cGMP) [56], which may counteract cAMP lowering by A1 receptor signaling.

The agonists and antagonists often also exhibit some side effects due to the role of A1 and A2 receptors in other signaling pathways, which has spawned attention to positive allosteric modulators (PAMs) [50,53,58,59,60]. Like all allosteric regulators, PAMs bind to a regulatory site of the receptor (GPCR) and alter the structure of the latter. The temporal and spatial specificity of a PAM is based on the principle that they come into play only when adenosine levels increase, at which time the PAM amplifies adenosine’s action at a particular AR subtype. It has been noted that the receptor selectivity of PAMs is due to the fact that their GPCR-binding regions (Figure 4) are non-canonical and highly diverse, which minimizes binding to the GPCRs of noncognate receptors [50,60]. For the record, PD81723 (Figure 6), a specific PAM for A1 receptor agonists, was the first successful PAM reported [56]. The fervent activity in this area has led to the chemical synthesis of a large number of PAMs of both A1 and A2A, while more are being routinely made and tested [50].

### 5.2. Intervention via the GPCR of Vagus Nerve NPY2R

As described before, definitive experiments in mice have shown that the vagus nerve family member NPY2R plays a cardinal role in syncope (Section 3.1). Like the adenosine receptor family, the NPY2R also is a Class A GPCR that signals through Gαi proteins to inhibit cAMP production. The cryo-electron microscopy of NPY2R with bound peptides derived from neuropeptide Y [24] has revealed the molecular basis of their specificity to stimulate NPY receptor type 2 but not type 1; however, extensive research on synthetic agonists of NPY receptors is needed to find a compound or peptide agonist that is optimal for pharmacological intervention. Ironically, although the type 2 receptor has been demonstrated to be important in syncope [1], it remains known whether its natural agonist, i.e., the neuropeptide Y, has any role in this process.

Lastly, in cases where persistent syncope is due to a chronically overactive neuron, its surgical removal, known as ‘neuroablation’, has produced dramatic relief [61]. Post-procedural heart rate response is usefor for predicting syncope recurrence or positive head-up tilt table testing after cardioneuroablation [61,62]. Evidently, further study of this promising approach is likely to further its use in curing vasovagal syncope [63,64].

## 6. Summary and Conclusions

**Figure 7:**
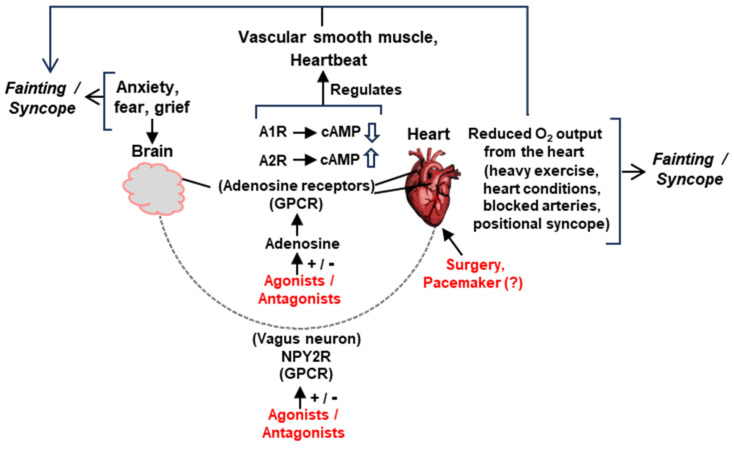
Schematic summary of the major pathways of signaling and suggested interventions (details in various Sections). The two main organs in syncope/fainting, namely, the brain and the heart, are physically joined by the vagus nerve, endowed with the neuropeptide Y receptor 2 (NPY2R). Various tissues in this network also contain adenosine receptors, A1R/A2R. Both NPY2R and A1/A2Rs are seven-helix GPCRs and can be manipulated by agonists, antagonists, or allosteric modulators. Manipulation of cyclic AMP (cAMP) synthesis is also a major mechanism by which the A1/A2Rs function. The regulation of ion channels is not shown for space constraints. Suggested representative interventions are in red. Myriads of other factors can precipitate syncope (such as head trauma, fear of heights, and other emotional factors), which may or may not lower the cardiac output. Head injury, for instance, may produce a constitutively overactive vagus nerve, prone to promote an occasional syncope or constant low-grade vertigo (our unpublished observation). The structural defects of the heart, vagus nerve, or vasculature—whether congenital or acquired—can only be relieved by surgical intervention. Implantable drug delivery devices, which may also be user activated when syncope is felt to be imminent, remain an untested option not depicted here.

Here, we first provide a schematic of our current knowledge of syncope (Figure 7).

## 7. Future Directions

As commented previously, the mechanisms of syncope have long been a puzzle, in part because most researchers generally study either the heart or the brain and not the inter-organ communications. The NPY2R study in the laboratory mouse was the first to fill this gap. Based on all the foregoing, we can propose several future directions of research on syncope, as listed below.

(i) Identify vertigo-/syncope-prone individuals in the human population and determine the sequence of their NPY2R to detect differences from the clinically normal cohorts. Pursue hereditary patterns and SNPs in a wider population-genetic survey. These studies may also unravel other genes, the products of which interact with NPY2R [also see (iii) below]. The methodology of such analyses is well established and feasible [65,66].

(ii) Compare human and mouse NPY2R orthologs so that the human amino acids can be introduced in transgenic mice and the behavior of the chimera studied. A genome-wide comparison of human and mouse G protein receptors carried out several years ago found that 56 mouse GPCRs had human orthologs [25]. It is tempting to speculate that the NPY receptor is one of them.

(iii) Recently, interactome mapping of several neurodegenerative diseases connected ∼5000 human proteins via ∼30,000 candidate interactions, linking many proteins known for causing neurodegenerative diseases, such as α-synuclein, TDP-43 (TAR DNA-binding protein 43), and ATXN1 (Ataxin 1), and revealing interconnectivity across this family of diseases [67,68]. It is entirely possible that such interactomes can be discovered for NPY2R, which may reveal its interacting partners among the brain and heart proteins, specifically those located in the syncope-relevant areas, namely, the medulla oblongata of the brain and the ventricle wall of the heart. Previously, several GPCRs were also located in specific areas of the brain [25]. This will allow more specific interventions of fainting, using trigger-point injections, for example.

(iv) A highly biased antagonist of the overactive NPY2R VSN would be an ideal treatment for syncope. Controlled delivery of electrical impulses to the nerve is already in use for certain types of epilepsy, cluster headaches, depression, and stroke rehabilitation, although not specifically for syncope per se [69,70,71,72,73]. The parameters of the treatments can be customized for each individual.

(v) Properly designed chimeric ligands and allosteric modulators of GPCRs have been shown to exhibit dual specificity [74,75] and offer several advantages over monospecific agonists [24,50]. It is worth asking if an adduct between the neuropeptide and A1 agonist functions for both receptors and exhibits improved pharmacokinetics.

Finally, syncope/fainting is a major public health burden. It has been estimated that sometime in their lives, 40% of adults will have syncope, and many will be admitted to the hospital, some in the emergency departments. The average length of stay is two days, and the average cost per admission in 2011 was USD 28,000 [76]. Recurrent syncope admission rates are also high, and the diagnosis and workup are highly reproducible for such cases, offering hope that the prevention regime, once optimized for an individual, will remain effective for multiple episodes. Medical devices, however, have remained scanty, but pacemaker-like devices have been described to benefit patients with dysautonomia [77]. If rigorously optimized, they may be adapted for use in syncope/fainting.
